# Childhood leukaemia incidence around French nuclear installations using geographic zoning based on gaseous discharge dose estimates

**DOI:** 10.1038/sj.bjc.6603111

**Published:** 2006-04-18

**Authors:** A-S Evrard, D Hémon, A Morin, D Laurier, M Tirmarche, J-C Backe, M Chartier, J Clavel

**Affiliations:** 1 INSERM, U754, Villejuif, France; 2 Université Paris Sud, IFR69, Villejuif, France; 3 Institut de Radioprotection et de Sûreté Nucléaire, IRSN/DRPH/SER/UETP, Fontenay-aux-Roses, France; 4 Institut de Radioprotection et de Sûreté Nucléaire, IRSN/DRPH/SRBE/LEPID, Fontenay-aux-Roses, France

**Keywords:** childhood leukaemia, nuclear sites, incidence, estimated dose

## Abstract

The present study investigated for the first time the incidence of childhood leukaemia (1990–2001) around French nuclear installations using a geographic zoning based on estimated doses to the red bone marrow due to gaseous radioactive discharges. The observed number of cases of acute leukaemia (*O*=750) in 40 km^2^ centred on 23 French nuclear installations between 1990 and 2001 was lower than expected (*E*=795.01), although not significantly so (standardised incidence ratio SIR=0.94, 95% confidence interval=(0.88–1.01)). In none of the five zones defined on the basis of the estimated doses was the SIR significantly >1. There was no evidence of a trend in SIR with the estimated doses for all the children or for any of the three age groups studied. This study confirmed that there was no evidence of an increased incidence of childhood leukaemia around the 23 French nuclear sites.

The epidemiological studies of leukaemia around nuclear sites have analysed incidence with respect to the distance from the plants, without considering details on the levels or geographic distribution of the radiation dose due to discharges from the plants (Laurier and Bard, 1999; Laurier *et al*, 2002). Such an approach may lead to inappropriate grouping of areas studied, since the dispersion of radionuclides is generally not isotropic and does not decrease linearly with the distance, while the dose levels are not equal for all sites. The present study investigated for the first time the incidence of childhood leukaemia (1990–2001) around French nuclear installations using a geographic zoning based on estimated doses to the red bone marrow (RBM) due to gaseous radioactive discharges.

In France, the levels of environmental contamination are generally below detection limits of routine monitoring. Doses therefore have to be estimated from discharge monitoring data and mathematical modelling of radionuclide transfer in the environment. This approach is used for regulatory purposes, to estimate doses to critical groups of the population, in the context of the discharge authorisation procedure (Chartier *et al*, 2002). In this paper, the approach has been adapted in order to estimate the geographic distribution of doses due to gaseous discharges in 40 km^2^ centred on 24 nuclear installations in France and derive a spatial zoning based on dose level order of magnitude. Beyond 20 km from the sites, the doses become extremely low.

A previous study of the incidence of leukaemia around all French nuclear installations found neither evidence of an overall increased incidence of childhood leukaemia in the vicinity of the 29 main nuclear installations during 1990–1998 nor a trend in standardised incidence ratio (SIR) with increasing distance from the sites for all children under 15 years of age (White-Koning *et al*, 2004). Compared to this study, the present study includes three additional years of observation (1990–2001).

## SUBJECTS AND METHODS

### Observed numbers of cases

The cases were all children under 15 years of age diagnosed with leukaemia between 1st January 1990 and 31st December 2001 and living around nuclear sites at the time of diagnosis. The cases were provided by the French National Registry of Childhood Leukemia and Lymphoma, which registered 5330 cases of acute leukaemia from 1990 to 2001 for the mainland France (Clavel *et al*, 2004). Eighty-one per cent of all the cases of acute leukaemia were acute lymphoblastic leukaemia (ALL) and 17% were acute myeloid leukaemia (AML). The incidence of acute leukaemia among children aged <15 years varies markedly with age (Clavel *et al*, 2004). In particular, ALL incidence shows a typical peak at age 2 years for girls and 3 for boys. Therefore, the analyses were also performed by age group (0–4, 5–9 and 10–14 years).

The Chooz and Civaux nuclear power plants were connected to the grid in 1997 and 1999, respectively. Therefore, the childhood leukaemia cases around those two sites were taken into account from 1997 for Chooz and 1999 for Civaux.

### Expected numbers of cases

Age- and gender-specific population counts by ‘*commune*’ (the smallest French administrative division), were derived from the national censuses of March 1990 and March 1999 provided by the National Institute for Statistics and Economic Studies (INSEE). A ‘*département*’ is an administrative geographic unit including 383 ‘*communes*’ on average. The annual number of births by gender for each ‘*commune*’ and the annual age- and gender-specific population estimates for each ‘*département*’ were available for each year from 1990 to 2001 (INSEE). They were used to obtain age- and gender-specific population estimates from 1991 to 1998 and for years 2000 and 2001 for each ‘*commune*’. The number of person years for a given year and a given ‘*commune*’ were subsequently calculated using those estimates. National age- and gender-specific incidence rates for childhood leukaemia in France (1990–2001), based on the National Registry data, were used as reference rates to derive annual expected numbers of cases for each age group and ‘*commune*’ near nuclear sites. The annual expected numbers of cases were used to estimate SIR defined as the ratio of the observed over the expected number of cases. The exact 95% confidence intervals (95% CI) for these ratios were given using a Poisson distribution.

## EXPOSURE ASSESSMENT

The spatial distribution of the exposure of the population around French nuclear installations due to gaseous radioactive discharge has been assessed by the Institute for Radiation Protection and Nuclear Safety (IRSN) using radionuclide discharge data, local climate data, and a mathematical model of nuclide transfers in the environment (Morin and Backe, 2002, 2003). Four types of installations were selected: nuclear power plants (NPP), nuclear fuel cycle plants (one fuel production facility, one site with a fuel conversion facility and a fuel enrichment facility), a nuclear fuel reprocessing plant, and two research centres. All the 19 French NPPs have been taken into account. They include two to six reactors ranging from 900 electric megaWatts (MW) to 1450 MW. For plants of other types (nuclear fuel cycle plants, nuclear fuel-reprocessing plant, research centres), only a few sites were selected to test the feasibility of the approach and its consistency with the available data. The selected nuclear fuel cycle plants include the nuclear fuel processing plant at Romans-sur-Isère and the fuel conversion plant and the fuel-enrichment plant at Pierrelatte. Two nuclear research centres at Saclay and Cadarache were selected together with the La Hague nuclear fuel-reprocessing plant. Previous dose calculation work for the population living near the La Hague reprocessing plant has been carried out in France (GRNC, 1999; Laurier *et al*, 2000; Rommens *et al*, 2000). Only the average dose delivered to the Beaumont-La Hague ‘*canton*’ (a geographic unit which, in this case, includes 10 ‘*communes*’) was determined. No zoning was carried out to differentiate the ‘*communes*’.

For the NPPs, the average annual discharge levels and discharge compositions for recent years were determined for each of the two types of NPP, namely the 900 MW and 1300 MW NPPs. The discharges from the 1450 MW Chooz and Civaux NPPs were assumed similar to the discharge from the 1300 MW NPPs. The typical composition of NPP gaseous discharges was taken into account. The composition includes the following nuclides: tritium, carbon 14, argon 41, krypton 85, xenon 133, xenon 135, iodine 131, iodine 133, cobalt 58, cobalt 60, caesium 134 and caesium 137. Carbon 14 was not measured in the gaseous effluent of the French NPPs until very recently. Therefore, no discharge data on carbon 14 were available, and the levels were assumed equivalent to the limit specified in recent discharge permits for this radionuclide. The average discharge levels and compositions for a period of several years (3- to 5-year period, depending on the data available) were assessed for the other plants. Those levels and compositions were assumed to be representative of discharges for recent years (after 1995) and have been used as input data in the dose estimations. Local climate data on wind speed and direction, vertical stability of the atmosphere and rain frequency were used for each site when available. Data were collected from the documents submitted by operators in discharge authorisation applications. For a few nuclear plants, data on vertical stability and on rain frequency were not available and the national average data were used.

The FOCON96 1.0 (Rommens *et al*, 1999) code was used to calculate the doses from routine discharge into the atmosphere. This code includes a model of gas and aerosol dispersion in the atmosphere. The model is based on a Gaussian model with the modelling of vertical and horizontal standard deviation developed by Doury (1976). The code also includes models of dry and wet deposition of aerosols on soil, grass and vegetable leaves. Wet deposition is based on a model of plume scavenging by the rain. The code takes into account root absorption of nuclides by vegetables and grass. Contamination of meat and milk is also modelled in the code. The default values of the transfer parameters proposed in the FOCON 96 code have been used. The main pathways have been taken into account: inhalation, ingestion (vegetables, meat and milk), external exposure from the plume (in the atmosphere or in the water) and deposits (aerosol deposition on the ground and sediment deposition on beaches or river banks). Protection by buildings was not taken into account in the assessment of external exposure. National average food consumption rates have been taken into account (INSEE, 1991). Only estimates of the local part of food production were taken into account (from national average data). It was assumed that 100% of the year was spent in the ‘*commune*’. The dose coefficient to RBM calculated by the International Commission of Radiological Protection (2002) was used for internal pathways and the US Federal Guidance dose coefficient for external exposure (Eckerman and Ryman, 1993).

Red bone marrow doses from gaseous discharge were estimated around each site on a polar grid (252 assessment points) around the stack with the FOCON 96 code. The doses were interpolated on a 250-m^2^ mesh using a SPLINE method (G3GRID procedure of the SAS^©^ software). The RBM dose for each ‘*commune*’ is the average of the four nearest mesh points around the town-hall of the ‘*commune*’.

### Areas under study

The studied area was defined as all ‘*communes*’ located in 40 km^2^ centred on 24 French nuclear installations. There are 36 354 ‘*communes*’ in France with an average population per ‘*commune*’ of 1609 people. A total of 2107 French ‘*communes*’ were included in the study. A strict partition of the areas under study had to be maintained in order to ensure the statistical independence of the observations. Therefore, when study areas around two sites overlapped (this occurred for 68 ‘*communes*’), the ‘*communes*’ were assigned to the site for which the estimated RBM dose was the highest. The total RBM dose obtained by adding the RBM dose estimates for the two sites was then assigned to the ‘*commune*’ considered. Since the Tricastin NPP and Pierrelatte plant are very close to each other, they were considered as a single site throughout the study. This explains the reference to 23 sites (18 NPPs) rather than the original 24 sites (19 NPPs). All of the 2107 ‘*communes*’ were subsequently divided into five zones defined on the basis of the estimated dose. Each of the lowest two categories of estimated dose included approximately a tertile of the expected number of cases in order to obtain stable incidence estimates in each category. The corresponding arithmetic means were 0.021 and 0.057 microSievert per year (*μ*Sv y^−1^) , respectively. In order to cover the full range of variation of the estimated dose, the third category was then divided into three categories using a logarithmic scale. The corresponding arithmetic means were 0.141, 0.553 and 2.13 *μ*Sv y^−1^, respectively. Each of the five categories was constructed as aggregations of the ‘*communes*’ whose estimated dose was within the limits of the category.

### Statistical analysis

The present study investigated for the existence of an increase in the SIR of childhood leukaemia with increasing estimated radiation dose due to gaseous discharge from nuclear sites. The following four tests were used: Fisher's *χ*^2^ test, the likelihood ratio test, a linear risk score test, and Stone's Poisson maximum test. Fisher's *χ*^2^ test and the likelihood ratio test based on the Poisson regression models were used to examine the heterogeneity of the five predefined categories of estimated dose. The linear risk score test and Stone's Poisson maximum test explicitly investigate for an increase in SIR with increasing estimated dose. The linear risk score test used was adapted from those used by Bithell *et al* (1994) and discussed by Bithell (1995): for the test, each case is scored on the basis on the estimated dose of the ‘*commune*’ under consideration. The Stone's Poisson maximum test is based on the maximum value of the SIR as ‘*communes*’ ranked by increasing estimated dose are aggregated into a region of greater size (Stone, 1988). The latter two tests were applied to the five predefined dose-based categories and to the estimated dose considered quantitatively for each ‘*commune*’. The powers of the linear risk score tests and Stone's Poisson maximum test to detect a given risk pattern have been discussed by several authors (Bithell, 1995; White-Koning *et al*, 2004; COMARE, 2005).

For all four tests, both an external and an internal reference were used. In the case of tests using an external reference, rejecting the null hypothesis (i.e. a uniform SIR of 1 irrespective of the estimated dose) might evidence the existence of a trend in the relative risk with the estimated dose, or might be due to an excess risk in the overall study area compared to the whole of France, which was considered as the external reference in the study. Using an internal reference enables only the distribution of cases within the study area to be considered and ignores the difference between the overall observed and expected numbers of cases around a given site.

The 5% critical values and the *P*-values of the four test statistics were estimated using simulation methods with R-software. Under the null hypothesis of a uniform SIR of 1 irrespective of the estimated dose, the null distributions of the four tests were determined from 10 000 simulations. The simulations were based on a Poisson distribution with expected values equal to the expected numbers of cases in the case of an external reference, or on a multinomial distribution with expected values proportional to the expected numbers of cases in the case of an internal reference. The 5% critical values and the *P*-values were then derived from the null distributions.

The analyses were performed for the 23 sites, for all cases (0–14 years) and for the complete period (1990–2001), and then separately, by age group (0–4, 5–9 and 10–14 years), period (1990–1995, 1996–2001), and leukaemia type (ALL, AML). The 18 NPPs were analysed as a separate subgroup because of their common characteristics. The possible heterogeneity of the 23 sites led to a more detailed study of each site individually. Bonferroni's method was used in order to correct for multiple testing.

## RESULTS

The estimated doses due to gaseous discharge in the 2107 ‘*communes*’ located in the vicinity of the 23 nuclear installations ranged from 0.06 to 1.33 *μ*Sv y^−1^, with an arithmetic mean of 0.17 *μ*Sv y^−1^, and a standard deviation of 0.48 *μ*Sv y^−1^.

Table 1 shows the distribution of the observed and expected numbers of childhood leukaemia cases and the SIRs by the above dose-based categories, for each of the 23 nuclear sites, for the 18 NPPs considered together and for the 23 sites considered together. The observed number of cases of childhood leukaemia within the study area (*O*=750) was lower than expected (*E*=795.01), but the difference was not statistically significant (SIR=0.94, 95% CI=[0.88–1.01]). Considering all 18 NPPs or all 23 sites, no evidence of heterogeneity between the five dose-based categories, and no trend toward an increase in SIR with increasing estimated dose, considered qualitatively or quantitatively, were found.

The likelihood ratio test and Fisher's *χ*^2^ test showed significant heterogeneity of the 23 nuclear sites (*P*=0.025 and 0.027, respectively). A statistically significant excess of cases was observed for one plant (Chinon: *O*=20, SIR=1.83, 95% CI=[1.12–2.83]), and a statistically significant deficit of cases was found for two plants (Fessenheim: *O*=17, SIR=0.50, 95% CI=[0.29–0.80]; Pierrelatte/Tricastin: *O*=6, SIR=0.41, 95% CI=[0.15–0.90]). When Bonferroni's method was used to correct for multiple testing (23 comparisons), neither the excess nor the deficits remained significant.

The overall tests are largely influenced by the Saclay site, which is located close to Paris and hence includes most of the cases. Therefore, the main analyses were also carried out on the 22 remaining sites excluding Saclay. There were 292 cases of childhood leukaemia compared to an expected number of 310.88 (SIR=0.94, 95% CI=[0.83–1.05]). No evidence of either heterogeneity between the five dose-based categories or a trend toward an increase in SIR with increasing estimated dose considered qualitatively or quantitatively, was found.

None of the 23 sites showed any statistical evidence of a SIR trend with increasing estimated dose, or of heterogeneity between the five dose-based categories. A likelihood ratio test based on Poisson regression models did not show any statistically significant heterogeneity for the slopes of the relationship between estimated dose and childhood leukaemia incidence estimated for each of the 23 sites separately (*P*=0.46). The common estimation of these slopes was not significantly different from 0: exp*β̂*=0.83 per *μ*Sv y^−1^ (95% CI=[0.58–1.20]) where *β̂* is the regression coefficient associated to the estimated dose.

Table 2 shows the distribution of the observed and expected numbers of childhood leukaemia cases and the SIRs by category of estimated dose for age groups 0–4, 5–9 and 10–14 years. No evidence of either heterogeneity between the five dose-based categories or a trend towards an increase in SIR with increasing estimated dose considered qualitatively or quantitatively, was found for any of the three age groups, when all 18 NPPs, all 23 sites or only 22 sites were considered.

There was no statistical evidence of heterogeneity between the five dose-based categories or of a trend towards an increase in SIR with increasing estimated dose, considered qualitatively or quantitatively, for any of the periods (1990–1995, 1996–2001), or for any of the leukaemia types (ALL, AML), when all 18 NPPs or all 23 sites were considered.

## DISCUSSION

The observed number of cases of acute leukaemia (*O*=750) in 40 km^2^ centred on the 23 French nuclear installations between 1990 and 2001 was lower than expected (*E*=795.01), but not significantly so (SIR=0.94, 95% CI=[0.88–1.01]). In none of the five zones defined on the basis of the estimated dose due to gaseous discharges was the SIR significantly greater than 1.0. There was no evidence of an increasing trend in SIR with increasing estimated dose for all children or for any of the three age groups studied.

The present study focused on developing a zoning method based on dose level order of magnitude using the same model for all nuclear installations. Inaccurate dose estimation cannot be excluded but since the same methodology was used for all sites, if the model or generic parameters were erroneous, the estimated doses would be uniformly raised or reduced; the geographic zoning would thus remain similar and the trend results would be unchanged. Conversely, inaccuracies related to the characteristics of each site, especially those related to local climate data, may have resulted in geographic zoning. However, climatologic data are derived from multiyear measurements and are not likely to be significantly inaccurate. The geographic zoning derived from dose estimates used in the present study is therefore considered not to be seriously erroneous. The dose estimates were based on a simple method. In particular, discharge has been assumed to occur at ground level for NPPs because the stacks are only a few meters higher than buildings (wake effect on the plume). For NPPs, insufficient data on the real discharge rates of carbon 14 were available. In consequence, relatively high rates for the discharge of carbon 14 by NPPs were assumed: the regulatory limits for discharge rates. This is likely to overestimate the contribution of carbon 14 to the estimated dose. The doses were estimated for adults because a sensitivity analysis showed that the estimated dose was rather similar for all the ages considered. The method of dose estimation was based on recent discharge rate data from the late nineties. As discharges generally show a sharp decrease with time, these data may lead to an underestimation of the doses compared to those associated to past discharges. On the other hand, the data are characteristic of the period 1996–2000, which approximately corresponds to the period of leukaemia diagnosis (1990–2001).

Further contributions to the total dose of radiation may be taken into account from direct radiation and liquid discharges. Direct radiation is minor around the French nuclear installations and can be neglected. Doses due to liquid discharges are possibly of the same order of magnitude as those from gaseous discharges, but may be considered homogeneous at the geographic scale used for gaseous discharges. Liquid-discharge doses depend on factors that are not subject to local borders, that is agricultural habits, food harvesting and distribution channels, and individual behaviour (bathing, fishing, consumption of food contaminated by irrigation from waterways). In consequence, the doses from liquid discharges could not be estimated in the present study, but the doses from gaseous discharges are considered to classify ‘*communes*’ correctly. Moreover, the results of the analyses were unchanged when the type of environment (sea, river, pool, etc.) of the nuclear sites under study was taken into account.

The epidemiological studies of the incidence of leukaemia around nuclear sites analysed incidence as a function of the distance from the sites. However, the dispersion of the radionuclides is generally not isotropic, the estimated dose does not decrease linearly with the distance and dose levels are not equal for all sites. The method of zoning used in the present study enables estimation of the real geographic distribution of the carcinogen dose (ionising radiation) in the environment (Morin and Backe, 2002, 2003). The zones defined by dose assessments were very different from concentric circles around the plants due to topographic and meteorological characteristics (Morin and Backe, 2002, 2003). The estimated dose and distance were significantly negatively correlated (Spearman's rank correlation coefficient *ρ*=−0.58, *P*<10^−4^), but marked variability in the estimated dose within each concentric band (0–5, 5–10, 10–15, 15–20 km) remained. The contrast in the mean dose between the lowest and highest dose-based categories (range: 2.11 *μ*Sv y^−1^; ratio: 106) was much larger than the maximum contrast between the concentric bands 0–5 and 15–20 km (range: 1.16 *μ*Sv y^−1^; ratio: 30).

The estimated dose attributable to gaseous discharge delivered to the population was very small for all ‘*communes*’ located in 40 km^2^ centred on the nuclear sites considered (mean dose <0.20 *μ*Sv y^−1^ and maximum value about a few microSievert per year). According to current knowledge on the effects of exposure to ionising radiation, no observable effect associated to such low doses is expected (UNSCEAR, 2000). The absence of excess risk or trend associated to the doses due to routine discharge from the plants is not surprising. In comparison, the RBM dose due to natural sources of exposure (including radon, terrestrial and cosmic gamma radiation, and intake of natural radionuclides) (Billon *et al*, 2005; Evrard *et al*, 2005, 2006) has been estimated to be 2700 *μ*Sv y^−1^ for children, and the dose due to medical examinations has been estimated to be approximately 740 *μ*Sv y^−1^ (Rommens *et al*, 2001). The mean estimated RBM dose due to gaseous radioactive discharge for children living in the vicinity of nuclear installations is therefore approximately 1000 to 10 000 times lower than the mean RBM dose due to natural sources.

## CONCLUSION

Most of the epidemiological studies of the incidence of leukaemia around nuclear sites analysed incidence as a function of the distance from the sites. In the present study, a dose-based zoning, rather than distance, was used for the first time in order to enhance the characterisation of the population's exposure. This approach has the advantage of considering the nonisotropic distribution of gaseous discharges in the environment. It also provides an illustration of the weakness of the doses due to routine discharges compared to other sources of exposure to ionising radiation. No evidence was found for a general increase or trend in the incidence of childhood leukaemia according to this zoning in the vicinity of the 23 French nuclear installations considered for the period 1990–2001.

## Figures and Tables

**Table 1:** Distribution of observed (*O*) and expected (*E*) numbers of childhood leukaemia cases by category of estimated dose due to gaseous discharge in the vicinity of each of 23 nuclear sites in France (1990–2001)

		**Estimated dose due to gaseous release (*μ*Sv y^−1^)**											
	**No**	**<0.045**		**0.045–0.072**		**0.072–0.316**		**0.316–1.0**		**⩾1.0**		**Total**	
**Nuclear sites (years^a^)**	**COM^b^**	***O***	***E***	***O***	***E***	***O***	***E***	***O***	***E***	***O***	***E***	***O***	***E***
*Nuclear power plants:*													
Belleville (1987)	58	1	0.79	2	0.63	3	2.82	0	0.34	0	0.11	6	4.68
Bugey (1979)	132	9	8.46	6	5.11	15	10.70	1	1.11	0	0.39	31	25.77
Cattenom (1986)	122	4	1.48	1	1.92	13	14.83	6	8.69	0	0.58	24	27.50
Chinon (1987)	88	9	4.85	1	1.11	7	3.30	3	1.31	0	0.32	20	10.90
Chooz (1997)	22	1	0.52	0	0.30	1	0.51	0	0.07	0	0.03	2	1.44
Civaux (1999)	58	3	0.99	0	0.19	0	0.51	0	0.03	0	0.02	3	1.75
Cruas (1983)	93	7	4.83	1	0.43	4	2.69	2	1.98	2	3.34	16	13.26
Dampierre (1980)	51	2	0.77	0	1.50	4	3.12	0	0.49	0	0.15	6	6.02
Fessenheim (1977)	69	17	31.86	0	0.53	0	1.35	0	0.20	0	0	17	33.94
Flamanville (1985)	27	0	0.43	0	0.57	0	0.36	2	0.54	0	0.32	2	2.22
Golfech (1990)	110	1	2.32	1	3.57	2	3.40	1	0.52	0	0.54	5	10.35
Gravelines (1980)	63	7	12.05	2	1.35	19	18.75	1	4.66	1	1.49	30	38.30
Le Blayais (1981)	89	1	0.72	0	1.69	5	3.34	1	1.09	0	0	7	6.84
Nogent (1987)	121	2	2.84	0	2.23	1	1.78	2	0.90	0	0	5	7.75
Paluel (1984)	127	0	1.34	1	0.98	4	4.66	0	0.50	0	0.54	5	8.01
Penly (1990)	133	7	4.20	3	1.81	5	5.36	0	0.36	0	0.33	15	12.06
St Alban (1985)	143	16	16.18	1	1.62	8	7.84	3	2.28	0	0.32	28	28.24
St Laurent (1981)	77	12	7.87	3	2.47	4	3.08	1	0.47	0	0.11	20	13.99

Total NPPs	1583	99	102.49	22	28.03	95	88.39	23	25.54	3	8.58	242	253.03
SIR (95%CI)		0.97 (0.79–1.18)		0.78 (0.49–1.19)		1.07 (0.87–1.31)		0.90 (0.57–1.35)		0.35 (0.07–1.02)		0.96 (0.84–1.08)	
													
Pierrelatte/Tricastin (1980)	78	2	4.10	0	1.66	3	6.26	1	2.45	0	0	6	14.47

Total NPPs+Pierrelatte/Tricastin	1661	101	106.59	22	29.69	98	94.65	24	27.99	3	8.58	248	267.49
SIR (95% CI)		0.95 (0.77–1.15)		0.74 (0.46–1.12)		1.04 (0.84–1.26)		0.86 (0.55–1.28)		0.35 (0.07–1.02)		0.93 (0.82–1.05)	

*Other sites:*													
Cadarache (1963)	47	7	6.18	1	0.90	3	1.52	0	0.14	0	0	11	8.74
La Hague (1967)	43	0	0	0	0	0	0	10	8.15	3	1.81	13	9.97
Romans-sur-Isère (1962)	114	20	24.68	0	0	0	0	0	0	0	0	20	24.68
Saclay (1950)	242	114	124.88	238	238.45	102	114.96	3	5.32	1	0.52	458	484.13

Subtotal (NPPs and other except Saclay)	1865	128	137.45	23	30.59	101	96.16	34	36.28	6	10.39	292	310.88
SIR (95% CI)		0.93 (0.78–1.11)		0.75 (0.48–1.13)		1.05 (0.86–1.28)		0.94 (0.65–1.31)		0.58 (0.21–1.26)		0.94 (0.83–1.05)	

Total (NPPs and other)	2107	242	262.34	261	269.04	203	211.12	37	41.60	7	10.91	750	795.01
SIR (95% CI)		0.92 (0.81–1.05)		0.97 (0.86–1.10)		0.96 (0.83–1.10)		0.89 (0.63–1.23)		0.64 (0.26–1.32)		0.94 (0.88–1.01)	

95% CI=95% confidence interval for the SIR; SIR=standardized incidence ratio=*O*/*E*.

a First year of operation

b Number of ‘*Communes*’.

**Table 2:** Distribution of observed (*O*) and expected (*E*) numbers of childhood leukaemia cases by age and by category of estimated dose due to gaseous discharge in the vicinity of 23 nuclear sites in France (1990–2001)

**Estimated dose (*μ*Sv y^−1^)**	**<0.045**	**0.045–0.072**	**0.072–0.316**	**0.316–1.0**	**⩾1.0**	**Total**
*0–4 years*						
O	111	149	110	19	5	394
E	134.60	145.01	109.39	20.69	5.38	415.08
SIR	0.82	1.03	1.01	0.92	0.93	0.95
95% CI	(0.68–0.99)	(0.87–1.21)	(0.83–1.21)	(0.55–1.43)	(0.30–2.17)	(0.86–1.05)

*5–9 years*						
O	72	71	52	6	1	202
E	76.81	75.63	61.02	12.49	3.27	229.22
SIR	0.94	0.94	0.85	0.48	0.31	0.88
95% CI	(0.73–1.18)	(0.73–1.18)	(0.64–1.12)	(0.18–1.05)	(0.01–1.70)	(0.76–1.01)

*10–14 years*						
O	59	41	41	12	1	154
E	50.93	48.40	40.71	8.42	2.25	150.71
SIR	1.16	0.85	1.01	1.42	0.44	1.02
95% CI	(0.88–1.49)	(0.61–1.15)	(0.72–1.37)	(0.74–2.49)	(0.01–2.47)	(0.87–1.20)

95% CI=95% confidence interval; SIR=standardized incidence ratio=*O*/*E*.
